# Regulation of FeLV-945 by c-Myb binding and CBP recruitment to the LTR

**DOI:** 10.1186/1743-422X-1-3

**Published:** 2004-09-03

**Authors:** Samantha L Finstad, Sudha Prabhu, Karen R Rulli, Laura S Levy

**Affiliations:** 1 Department of Microbiology and Immunology, Program in Molecular and Cellular Biology and Tulane Cancer Center, Tulane University Health Sciences Center, New Orleans, Louisiana, USA; 2 Science Applications International Corporation, Frederick, Maryland, USA

## Abstract

**Background:**

Feline leukemia virus (FeLV) induces degenerative, proliferative and malignant hematologic disorders in its natural host, the domestic cat. FeLV-945 is a viral variant identified as predominant in a cohort of naturally infected animals. FeLV-945 contains a unique sequence motif in the long terminal repeat (LTR) comprised of a single copy of transcriptional enhancer followed by a 21-bp sequence triplicated in tandem. The LTR is precisely conserved among independent cases of multicentric lymphoma, myeloproliferative disease and anemia in animals from the cohort. The 21-bp triplication was previously shown to act as a transcriptional enhancer preferentially in hematopoietic cells and to confer a replicative advantage. The objective of the present study was to examine the molecular mechanism by which the 21-bp triplication exerts its influence and the selective advantage responsible for its precise conservation.

**Results:**

Potential binding sites for the transcription factor, c-Myb, were identified across the repeat junctions of the 21-bp triplication. Such sites would not occur in the absence of the repeat; thus, a requirement for c-Myb binding to the repeat junctions of the triplication would exert a selective pressure to conserve its sequence precisely. Electrophoretic mobility shift assays demonstrated specific binding of c-Myb to the 21-bp triplication. Reporter gene assays showed that the triplication-containing LTR is responsive to c-Myb, and that responsiveness requires the presence of both c-Myb binding sites. Results further indicated that c-Myb in complex with the 21-bp triplication recruits the transcriptional co-activator, CBP, a regulator of normal hematopoiesis. FeLV-945 replication was shown to be positively regulated by CBP in a manner dependent on the presence of the 21-bp triplication.

**Conclusion:**

Binding sites for c-Myb across the repeat junctions of the 21-bp triplication may account for its precise conservation in the FeLV-945 LTR. c-Myb binding and CBP recruitment to the LTR positively regulated virus production, and thus may be responsible for the replicative advantage conferred by the 21-bp triplication. Considering that CBP is present in hematopoietic cells in limiting amounts, we hypothesize that FeLV-945 replication in bone marrow may influence CBP availability and thereby alter the regulation of CBP-responsive genes, thus contributing to altered hematopoiesis and consequent hematologic disease.

## Background

Feline leukemia virus (FeLV) is a simple gammaretrovirus that induces degenerative, proliferative and malignant hematologic disorders in its natural host, the domestic cat. Like other natural retroviruses, FeLV is not a single genomic species but is a genetically complex family of closely related viruses subject to selective pressures in the host. Variant genomes are generated during virus replication as a result of both error-prone reverse transcription and recombination. The consequence of this variation is a diverse population that is continuously shaped *in vivo *and from which variants with selective advantages arise as predominant species. The variable clinical outcome of FeLV infection is thought to reflect this genetic diversity [1,2]. FeLV-945, a natural FeLV variant, was originally identified as the predominant species in a temporal and geographic cohort of infected cats. FeLV-945 was originally derived from a multicentric lymphoma of unknown phenotype and subsequently identified in degenerative and proliferative diseases of myeloid and erythroid origin from the cohort. FeLV-945 contains a unique sequence motif in the long terminal repeat (LTR) comprised of a single copy of transcriptional enhancer followed 25-bp downstream by a 21-bp sequence triplicated in tandem. The sequence and position of the 21-bp triplication in the FeLV-945 LTR was observed to be precisely conserved among eight independent multicentric lymphomas and in cases of myeloproliferative disease and anemia in animals from the cohort [[3,4], Chandhasin *et al*., manuscript submitted].

The 21-bp triplication was previously shown to provide transcriptional enhancer function to the LTR that contains it, and to function preferentially in primitive hematopoietic cells [5]. In K-562 cells, a human leukemia cell line considered to be primitive and multipotential [6,7], the FeLV-945 LTR was 12-fold more active than other naturally occurring FeLV LTRs examined. Further, the FeLV-945 LTR was preferentially active in K-562 cells, 4.2-fold more active than in FEA feline embryo fibroblasts [5]. Interestingly, when the U3 region of the LTR containing the 21-bp triplication was placed downstream of a heterologous promoter, the preferential activity in K-562 cells was lost. These findings suggest that the ability of the 21-bp triplication to enhance transcription preferentially in hematopoietic cells depends on the presence of the adjacent LTR binding sites in their natural array, a possibility examined further in the present study. Previous studies also showed that the 21-bp triplication in the FeLV-945 LTR confers a replicative advantage to the virus that contains it, preferentially in hematopoietic cells [8]. This growth advantage may account for the induction of tumors of the type in which FeLV-945 was identified, and may represent a selective advantage that contributes to precise conservation of the unusual LTR sequence.

Regarding the molecular mechanism by which the 21-bp triplication functions in the context of the LTR, at least two possibilities have been considered. One possibility is that the 21-bp triplication functions to maintain the appropriate spacing in the LTR between the enhancer and the promoter. A spacer function might be particularly relevant in an LTR like FeLV-945 in which the enhancer is not tandemly repeated. Substitution of the 21-bp repeat element with unrelated sequence of the same length, however, was observed to ablate the replicative advantage, thus indicating that the 21-bp triplication does not perform solely a spacer function [8]. An alternative mechanism may be that the 21-bp triplication contributes genuine enhancer function, perhaps via the binding of nuclear transcription factors. Indeed, electrophoretic mobility shift assay demonstrated that the 21-bp triplication contains binding sites for specific nuclear proteins. These observations suggested that preserving the protein binding sites may confer a selective advantage that accounts for the precise sequence conservation of the 21-bp triplication in this natural FeLV isolate [8]. The present study examined this possibility further. Binding sites were identified for the transcription factor, c-Myb, that crossed the repeat junctions of the triplication. Further, once c-Myb was bound to the triplication, the transcriptional co-activator CBP was recruited and was shown to positively regulate virus production. Considering that CBP is present in hematopoietic cells in limiting amounts, these observations suggest that FeLV-945 replication in bone marrow may influence CBP availability and thereby alter the regulation of CBP-responsive genes, thus contributing to altered hematopoiesis and consequent hematologic disease.

## Results

**Figure 1:**
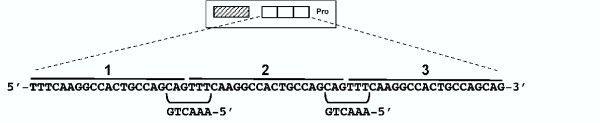
Diagram of the U3 region of the FeLV-945 LTR, indicating the transcriptional enhancer (hatched box), 21-bp triplication (open boxes) and transcriptional promoter (Pro). Below the diagram is shown the sequence of the 21-bp triplication, indicating putative binding sites for the c-Myb transcription factor formed across the repeat junctions. The c-Myb binding site consensus occurs in the negative strand.

**Figure 2:**
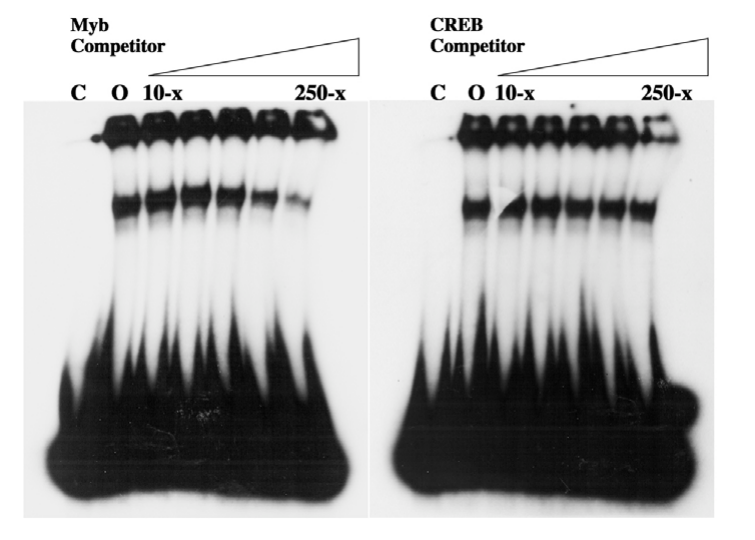
Electrophoretic mobility shift assays (EMSA) performed using a radiolabeled probe representing the 21-bp triplication from the FeLV-945 LTR. Nuclear extracts (3.5 μg) from K-562 cells were incubated with the radiolabeled probe (1 ng). Double-stranded competitor oligonucleotides were omitted from the reaction (lanes 0), or were included in increasing amounts from 10-fold to 250-fold molar excess (10-, 25-, 50-, 100- and 250-fold excess shown). The competitors used contained a c-Myb consensus binding site (5'-TACAGGCATAACGGTTCCGTAGTGA) or a CREB consensus binding site (5'-AGAGATTGCCTGACGTCAGAGAGCTAG). Also indicated is the migration of the radiolabeled probe without the addition of nuclear extract (lanes C).

**Figure 3:**
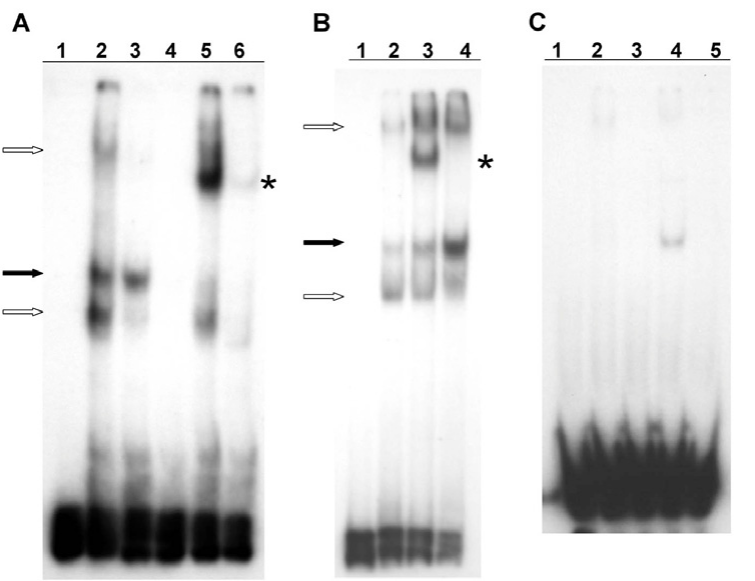
Supershift EMSA in the presence of a c-Myb-specific antibody. **(A)**. Nuclear extracts (5 μg) from K-562 cells were incubated with the radiolabeled GS945 probe (2.4 ng) representing the 21-bp triplication from the FeLV-945 LTR. Shown are probe only (lane 1), complex formation in the presence of nuclear extract (lane 2), and complex formation in the presence of 200-fold molar excess of non-specific (lane 3) or specific competitor (lane 4). Reaction performed in the presence of monoclonal antibody to c-Myb (4 μg) resulted in supershift of the specific complex (lane 5) which was not observed in the presence of 200-fold molar excess of specific competitor (lane 6). **(B)**. Lanes 1, 2 and 3 represent repetitions of lanes 1, 2 and 5 of (A). Reaction with a isotype control antibody (lane 4) did not result in supershift. Indicated are the specific complex (solid arrow), non-specific complexes (open arrows), and the supershifted complex (asterisk). **(C)**. EMSA performed using the radiolabeled GS61E probe, which contains only a single copy of the 21-bp element. Shown are probe only (lane 1), reaction performed in the presence of K-562 nuclear extract (5 μg; lane 2), and reaction performed in the presence of 100-fold molar excess of unlabeled GS945 (lane 3), GS61E (lane 4) or non-specific competitor (lane 5). The absence of complex formation using the GS61E probe demonstrates the requirement for the 21-bp triplication.

As described above, previous studies suggested that preserving the protein binding sites may confer a selective advantage that accounts for the precise sequence conservation of the 21-bp triplication in the FeLV-945 LTR [8]. In the present study, the sequence of the 21-bp triplication was compared to a transcription factor binding site database (TFSEARCH, based on TRANSFAC; [9]) in order to identify potential binding proteins. This analysis identified two putative binding sites for the transcription factor c-Myb formed across the repeat junctions of the triplication (Figure 1). The sequence of those sites, 5'-AAACTG, closely matched the consensus c-Myb binding sequence, YAACG/TG (Y = pyrimidine; [10,11]). Mismatch between the putative binding site and the consensus sequence was observed at position 1, a position whose change from T to A is known to have little effect on binding affinity [12]. To determine whether c-Myb binds to the FeLV-945 21-bp triplication, EMSA was performed by reacting a radiolabeled triplication-containing probe with nuclear extracts from K-562 cells in the presence of increasing amounts of a known high-affinity c-Myb binding site as competitor. K-562 cells were chosen because c-Myb is known to be expressed and is thought to be a regulator of their differentiation along multiple hematopoietic lineages [13]. The results demonstrated a significant reduction in complex formation in the presence of the c-Myb site competitor, especially at amounts in ≥ 100-fold molar excess. In contrast, 250-fold molar excess of the unrelated CREB binding site had no effect (Figure 2). To confirm the presence of c-Myb in the specific protein-DNA complex formed on the 21-bp triplication, supershift EMSA was performed using nuclear extracts from K-562 cells in the presence of a monoclonal anti-c-Myb antibody. The results clearly showed decreased mobility of the specific complex in the presence of the c-Myb antibody (Figure 3A), but not in the presence of an isotype control antibody (Figure 3B). As a control to confirm that c-Myb binding required repetition of the 21-bp element, EMSA was repeated with a homologous probe derived from FeLV-A/61E, a natural isolate that contains only a single copy of the 21-bp sequence in the LTR. The results demonstrated no specific complex formation on the FeLV-A/61E-derived probe (Figure 3C), confirming that the specific complex formed on the FeLV-945-derived probe is attributable to the 21-bp triplication.

**Figure 4:**
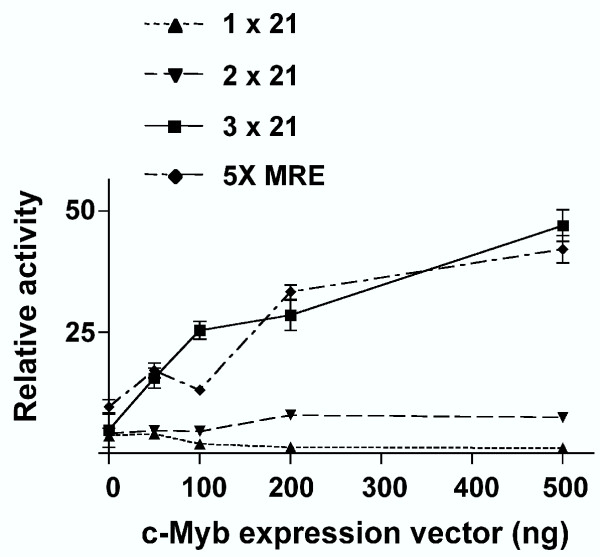
Response to exogenous c-Myb expression of FeLV LTRs containing variable numbers of the 21-bp element. Recombinant FeLV LTRs were constructed that contained 1, 2 or 3 copies of the 21-bp element and were cloned into a firefly luciferase reporter plasmid. LTR reporter plasmids or a 5X MRE positive control plasmid (500 ng) were introduced by lipid-mediated transfection in triplicate into feline embryonic fibroblasts (FEA) together with the *Renilla *luciferase reporter plasmid pRL-SV40 (5 ng) and a c-Myb expression plasmid in increasing concentrations (0 – 500 ng). Cell lysates were harvested 24 hours later and luciferase activity was quantified. Data are reported as a ratio of firefly to *Renilla *luciferase activity. Shown are data from a representative experiment repeated three times independently.

**Figure 5:**
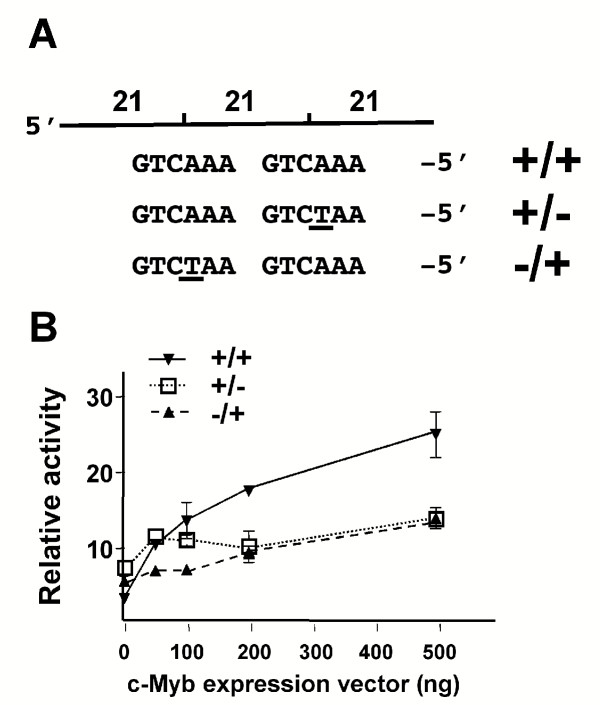
Response to exogenous c-Myb expression of FeLV LTRs containing c-Myb binding site mutations. **(A)**. Diagram of the 21-bp triplication as contained in the FeLV-945 LTR, indicating the sequence of c-Myb binding sites across the repeat junctions of the triplication (**+/+**). LTRs were constructed in which the first (**-/+**) or second (**+/-**) binding site was mutated. **(B)**. Firefly luciferase reporter gene plasmids containing the FeLV LTR with wild type or mutant c-Myb binding sites (500 ng) were introduced by lipid-mediated transfection in triplicate into feline embryonic fibroblasts (FEA) together with the *Renilla *luciferase reporter plasmid pRL-SV40 (5 ng) and a c-Myb expression plasmid in increasing concentrations (0 – 500 ng). Cell lysates were harvested 24 hours later and luciferase activity was quantified. Data are reported as a ratio of firefly to *Renilla *luciferase activity. Shown are data from a representative experiment repeated three times independently.

To evaluate whether c-Myb binding to the 21-bp triplication regulates LTR function, reporter plasmids were constructed in which expression of the firefly luciferase gene was driven by the U3 region of an FeLV LTR containing one, two or three copies of the 21-bp element. Reporter gene constructs were introduced by lipid-mediated transfection into feline embryonic fibroblasts (FEA) along with increasing amounts of a c-Myb expression vector. Fibroblasts were selected because the level of endogenous c-Myb expression in those cells is low or absent [14,15]. The results (Figure 4) demonstrated that the FeLV-945 LTR (3 × 21) responds to increasing levels of c-Myb expression to an extent statistically indistinguishable from the positive control, i.e., a reporter plasmid containing five tandem Myb-responsive elements (5X MRE). In contrast, an FeLV LTR containing only a single 21-bp element was unresponsive to c-Myb. The responsiveness of an LTR containing two 21-bp elements was also examined, since the 21-bp duplication would be predicted to encode one c-Myb binding site across the repeat junction. Interestingly, this LTR responded to increasing c-Myb expression to a low but statistically significant extent (p < 0.05 as compared to 1 × 21; Figure 4). These data show that the triplication-containing LTR is responsive to c-Myb in a dose-dependent manner and suggest that full responsiveness requires the presence of both c-Myb binding sites. To confirm the latter finding, a point mutation previously shown to ablate c-Myb binding [16] was introduced alternately into each of the sites (Figure 5A). Synthetic oligonucleotides containing the respective mutations were substituted into the LTR, and luciferase reporter gene constructs containing the mutant LTRs were introduced into FEA cells along with increasing concentrations of a c-Myb expression vector. LTRs in which either c-Myb binding site was ablated were observed to respond only weakly to increasing levels of c-Myb, and to significantly lower levels than the wild type LTR containing both binding sites (p < 0.05; Figure 5B).

Previous studies had shown that the 21-bp triplication contributes enhancer function to the LTR in a cell type-specific manner, and that it is significantly more active in K-562 cells as compared to a fibroblast line [5]. In hindsight, these results may be explained by the relatively high levels of c-Myb expression in K-562 cells and its relative absence in fibroblasts [14,15]. When the U3 region of the FeLV-945 LTR was placed downstream of a heterologous promoter, however, the cell type-specific preference for enhancer function was lost [5]. One explanation for these findings is that c-Myb bound to the 21-bp triplication may function through interactions with other proteins bound to the LTR, and that such interactions require the LTR binding sites to be present in their natural array. Indeed, c-Myb is known to function in a combinatorial manner with other transcription factors and co-activators to activate target gene expression [14]. Studies were performed in the present study to evaluate this possibility further. First, an oligonucleotide containing only the 21-bp triplication was cloned into a luciferase reporter plasmid upstream of a heterologous SV40 promoter. This construct, when introduced into FEA cells, was observed to be unresponsive to increasing levels of c-Myb expression (data not shown). Thus, the presentation of c-Myb binding sites through the 21-bp triplication is apparently insufficient to regulate transcription in the absence of the normally adjacent LTR enhancer and promoter. These findings are consistent with the possibility that c-Myb binding to the 21-bp triplication functions to activate transcription by interacting with proteins bound to adjacent sites on the LTR. c-Myb is known to interact directly with a number of different proteins, including the transcriptional co-activator CREB-binding protein (CBP) [14,15]. Indeed, CBP is thought to act as a bridge that physically connects c-Myb to the promoter-bound basal transcription machinery, thus stabilizing the transcription-preinitiation complex [14,15,17]. Experiments were therefore performed in the present study to examine the possibility that c-Myb bound to the 21-bp triplication interacts with CBP.

**Figure 6:**
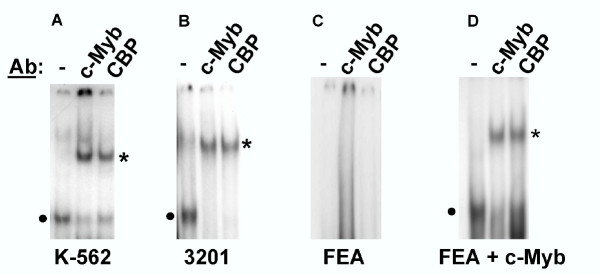
Supershift EMSA in the presence of antibody specific for c-Myb or CBP. **(A – C)**. Nuclear extracts (5 μg) from K-562, 3201 or FEA cells were incubated with the radiolabeled GS945 probe (2.4 ng) representing the 21-bp triplication from the FeLV-945 LTR. Shown in each panel is specific complex formation in the presence of nuclear extract (closed circle), and with the addition of monoclonal antibody to c-Myb or CBP (4 μg). Reduced mobility of the complex (supershift) is indicated (asterisk). **(D) **shows the same reactions performed with nuclear extracts from FEA cells in which c-Myb was exogenously overexpressed.

**Figure 7:**
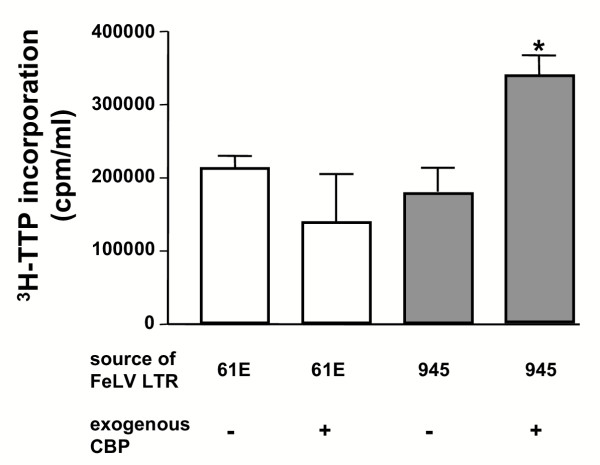
Regulation of FeLV replication in response to exogenous overexpression of CBP. K-562 cells were chronically infected with recombinant FeLV containing the LTR of FeLV-945 or FeLV-A/61E. A CBP expression plasmid was then introduced by lipid-mediated transfection. Culture supernatants were collected 3 days later and reverse transcription activity was quantified as a measure of virus production. Results are reported as cpm/ml of ^3^H-TTP incorporated. The data shown were pooled from two independent experiments each performed in triplicate.

Supershift EMSA was used to evaluate whether c-Myb and CBP might be present in the specific complex that forms on the 21-bp triplication. Specific complex formation was observed using a radiolabeled probe representing the 21-bp triplication in the presence of nuclear extracts from either K-562 cells or feline 3201 T-cells. In both cases, mobility of the complex was reduced (supershifted) when reacted with antibody to c-Myb. When the same reaction was performed in the presence of an antibody to CBP, an identical supershift was observed (Figure 6A,6B). No complex formation was observed when the probe was reacted with nuclear extracts from FEA cells (Figure 6C), consistent with the lack of c-Myb expression in fibroblasts [14,15]. CBP is ubiquitously expressed [14]; therefore, this observation indicates that CBP does not participate in complex formation on the 21-bp triplication in the absence of c-Myb. When c-Myb was expressed exogenously in FEA cells, specific complex formation and supershift were observed in the presence of antibody to either c-Myb or CBP (Figure 6D). Finally, analysis of FeLV-945 replication indicated a regulatory role for c-Myb binding and recruitment of CBP to the 21-bp triplication. K-562 cells were infected with recombinant FeLV [8] containing the LTR of either FeLV-945 or FeLV-A/61E, the latter having only a single copy of the 21-bp element. A CBP expression vector was introduced into cells chronically infected with either virus, and virus production was measured three days later by quantifying reverse transcriptase activity in the culture supernatants. The results showed significantly increased levels of production of virus containing the FeLV-945 LTR. In contrast, virus containing the FeLV-A/61E LTR was unaffected (Figure 7). These findings indicate that the 21-bp triplication in the LTR renders the virus responsive to the amount of available CBP.

## Discussion

The natural FeLV isolate, FeLV-945, was originally identified from lymphoid and other hematopoietic disorders in a geographic and temporal cohort. A unique 21-bp repeat motif in the FeLV-945 LTR was observed to be precisely conserved among animals in the cohort that exhibited malignant, proliferative or degenerative hematopoietic diseases of non-T-cell origin [[3,4], Chandhasin *et al*., manuscript submitted]. The 21-bp triplication was shown to enhance transcription from the FeLV LTR and to confer a replicative advantage to the virus, at least in part through the specific binding of unidentified nuclear proteins to the repeat motif [5,8]. In the present study, sequence analysis revealed two potential c-Myb binding sites formed across the repeat junctions of the 21-bp triplication (Figure 1). While the sequence of the potential binding sites (AAACTG) did not match the consensus c-Myb binding site precisely (YAACG/TG; Y = pyrimidine; [10,11]), the sequence was observed to be as closely related to the consensus binding site as are several sites in the HTLV-I LTR that are known to bind c-Myb [18,19]. Indeed, electrophoretic mobility shift assays indicated the specific binding of c-Myb to the 21-bp triplication by showing that a known high-affinity c-Myb binding site competed for DNA-protein complex formation but an unrelated site did not. It was noteworthy in these assays that significant competition for complex formation occurred only when the competitor was present at relatively high amounts (≥ 100-fold molar excess; Figure 2). By comparison, c-Myb binding to a consensus sequence in the *bcl*-2 promoter was shown to be effectively competed by 50-fold molar excess of a cold oligonucleotide carrying a high affinity c-Myb binding site [20]. A possible explanation for this difference may be that, while c-Myb can recognize a single consensus binding site such as that found in the competitor oligonucleotide we used, the natural recognition sites are generally found in multiple, closely aligned copies as in the 21-bp triplication. Thus, the affinity of binding to the triplication may be higher than to the competitor. It is further known that sequences flanking the consensus binding site may also be important in determining c-Myb binding affinity [21]. Electrophoretic mobility shift assays performed in the presence of an antibody to c-Myb confirmed the presence of c-Myb in the specific DNA-protein complex (Figure 3A,3B), and confirmed that repeat of the 21-bp element was required for complex formation (Figure 3C).

The c-Myb transcription factor is a critical regulator of gene expression, proliferation and differentiation in early hematopoietic progenitors [14,15,22] and has been exploited as a transcriptional regulator by many viruses that infect bone marrow cells [19,23-25]]. Considering that FeLV is known to replicate in the bone marrow [26,27], and that FeLV-945 infection was associated with various diseases of hematopoietic origin [Chandhasin *et al*., manuscript submitted], it is likely that the tropism of FeLV-945 *in vivo *included the hematopoietic progenitors in which c-Myb is expressed. Considering this possibility, we hypothesized that c-Myb may act as a transcriptional regulator of FeLV-945. In support of this hypothesis, reporter gene assays showed that an LTR containing the triplication was responsive to c-Myb in a dose-dependent manner (Figure 4), and that optimal responsiveness required the presence of both c-Myb binding sites (Figure 5). The identification of c-Myb binding sites that spanned the repeat junctions of the 21-bp triplication was particularly noteworthy because such sites would not occur in the absence of the repeat. Thus, a requirement for c-Myb binding to the repeat junctions of the triplication would exert a selective pressure to conserve its sequence precisely. Results indicated further that when c-Myb binds to the 21-bp triplication, it interacts with the transcriptional co-activator CBP, a critical regulator of normal hematopoiesis [17]. Identical electrophoretic mobility supershifts were observed when protein-DNA complexes were formed in the presence of antibody either to c-Myb or CBP, consistent with the hypothesis that both proteins are present in the same complex (Figure 6A,6B). The data further indicated that c-Myb recruits CBP to the 21-bp triplication, since no CBP-containing complex formation could be demonstrated unless c-Myb was also expressed (Figure 6C,6D). Finally, virus production was shown to be positively regulated by CBP in a manner dependent on the presence of the 21-bp triplication (Figure 7). These results indicated that the interaction between c-Myb and CBP is functional, and suggest that the c-Myb-mediated recruitment of CBP to the FeLV-945 LTR could be responsible for the previously reported replicative advantage conferred by the 21-bp triplication [8].

CBP and c-Myb are thought to activate target genes in hematopoietic progenitors through various mechanisms of interaction. One of those mechanisms involves a bridging function in which CBP links c-Myb with components of the basal transcription machinery, thereby establishing and/or stabilizing the transcription complex [14,15,17]. While the mechanism of interaction was not investigated in the present study, the bridging function is an intriguing possibility because it might explain the observed requirement of the 21-bp triplication for an intact LTR enhancer and promoter. Specifically, when the isolated 21-bp triplication was positioned upstream of a heterologous promoter, it did not confer responsiveness to exogenously supplied c-Myb (data not shown). Previous studies had similarly shown that the 21-bp triplication could not exert its influence when placed downstream of a heterologous promoter [5]. These observations indicated that transcriptional activation of the FeLV-945 LTR through c-Myb/CBP interaction requires that the LTR binding sites be present in their natural array. The possibility that CBP exerts its influence on the FeLV-945 LTR through a bridging function is significant because it implies that CBP acts stoichiometrically. CBP is known to be present in bone marrow cells in limiting amounts, playing a major role in hematopoiesis through competitive utilization on target promoters [17,28-31]]. Considering the competitive utilization of limiting amounts of CBP in hematopoiesis, its stoichiometric recruitment to the FeLV-945 LTR might interfere with CBP availability and thereby alter the regulation of CBP-responsive genes. Such alteration might then contribute to altered hematopoiesis and consequent hematologic disease.

## Conclusions

FeLV-945 contains a unique 21-bp triplication in the LTR, conserved among animals in a geographic cohort with multicentric lymphoma, myeloproiferative disease or anemia. Binding sites for the c-Myb transcription factor were identified across the repeat junctions of the 21-bp triplication. Optimal responsiveness of the FeLV-945 LTR to c-Myb was shown to require the presence of both c-Myb binding sites. Since the binding sites would not occur in the absence of the repeat, a requirement for c-Myb binding would be predicted to exert a selective pressure for conserving the 21-bp triplication precisely. c-Myb binding to the 21-bp triplication was shown to recruit CBP, a transcriptional co-activator essential for hematopoiesis and known to be present in limiting amounts. Interaction of c-Myb and CBP with the 21-bp triplication was shown to positively regulate virus production, and thus may be responsible for the replicative advantage conferred by the repeat sequence. Considering that CBP is present in hematopoietic cells in limiting amounts, we hypothesize that FeLV-945 replication in bone marrow may influence CBP availability and thereby alter the regulation of CBP-responsive genes, thus contributing to altered hematopoiesis and consequent hematologic disease.

## Methods

### Cell lines and viruses

K-562, a malignant multipotential human hematopoietic cell line, was obtained from the American Type Culture Collection (CCL-243) and was maintained in RPMI 1640 medium with 10% FBS. The FEA cell line, a continuous line of feline embryonic fibroblasts, was obtained from Dr. Jennifer Rojko and was grown in Eagle minimal essential culture medium supplemented with 10% fetal bovine serum (FBS), 0.1 mM non essential amino acids and 50 μg/ml gentamicin reagent solution (Invitrogen, Carlsbad, CA,). 3201 is an FeLV-negative thymic lymphoma cell line of feline origin [32] and was maintained in 50% Leibovitz L-15 medium/50% RPMI 1640 supplemented with 15% FBS. Infectious recombinant FeLVs GA-945L and GA-61EL were constructed from an infectious molecular clone of FeLV-B/Gardner-Arnstein into which was substituted the LTR of FeLV-945 or of FeLV-A/61E, respectively, between *EcoRV *and *Hinc II *restriction enzyme sites [5,8]. The FeLV-A/61E LTR was selected because it represents a naturally occurring isolate of FeLV typical of those horizontally transmitted among cats in nature, and it contains only a single copy of the 21-bp element [33].

### Electrophoretic mobility shift assays

A double-stranded oligonucleotide probe containing the 21-bp triplication and 40 bp of flanking sequence from the FeLV-945 LTR was radiolabeled using the synthetic oligonucleotide GS945 as template (5'- GCTGAAACAGCAGAAGTTTCAAGGCCACTGCCAGCAGTTTCAAGGCCACTGCCAGCAGTTTCAAGGCCACTGCCAGCAGTCTCCAGGCTCCCCAGTTGAC -3'), the filling primer (5'-CTGGTCAACTGGGGAGCCT-3') and the Klenow fragment of DNA polymerase (Invitrogen, Carlsbad, CA) to complete the duplex. A homologous probe containing only a single copy of the 21-bp element was similarly synthesized using the oligonucleotide GS61E as template (5'-GCTGAAACAGCAGAAGTTTCAAGGCCACTGCCAGCAGTCTCCAGG CTCCCCAGTTGAC-3'). Nuclear extract from K-562 cells was obtained from Active Motif (Carlsbad, CA). Nuclear extracts from FEA and 3201 cells were prepared using the Nuclear Extract Kit from Active Motif (Carlsbad, CA) according to manufacturer specifications. Nuclear extracts were also prepared from FEA cells following the lipid-mediated transfection (Lipofectamine Plus reagent; Invitrogen, Carlsbad, CA) of FL-Myb, a c-Myb expression vector in which full length murine c-Myb cDNA was inserted into the multiple cloning site of pcDNA3.1 (a gift of Dr. Linda Wolff, National Cancer Institute). DNA-protein binding reactions included 5 μg of nuclear extract and 2.4 ng of radiolabeled probe in a 20 μl reaction containing 1 mM Tris pH 7.5, 7.5 mM NaCl, 1 mM EDTA, 0.7% glycerol, 0.1 mM DTT and 2 μg poly(dI-dC). Reactions containing nuclear extracts from K-562 cells were incubated at 4°C for 30 minutes. Reactions containing nuclear extracts from 3201 or FEA cells were incubated at 30°C for 30 minutes. In some reactions, unlabeled probe was added to the reaction as a specific competitor, or HindIII/HaeIII-digested bacteriophage lambda DNA was included as non-specific competitor. Some reactions included as competitor a double-stranded oligonucleotide containing a known high-affinity c-Myb consensus binding site (5'-TACAGGCATAACGGTTCCGTAGTGA) or a CREB consensus binding site (5'-AGAGATTGCCTGACGTCAGAGAGCTAG). Protein-DNA complexes were resolved by 6% polyacrylamide gel electrophoresis in 0.25X TBE buffer (1X TBE buffer is 89 mM Tris base, 89 mM Boric acid and 2 mM EDTA). Gels were then dried at 80°C and exposed to radiographic film for varying periods of time. In some reactions, monoclonal antibody (4 μg) to either c-Myb or CBP, or isotype control antibody, was added after the 30-minute incubation period and incubated overnight at 4°C. Complexes were then resolved by 6% polyacrylamide gel electrophoresis as described above. The mouse monoclonal IgG1 antibody to c-Myb was raised against a recombinant protein corresponding to amino acids 500–640 of the human protein (Santa Cruz Biotechnology, Santa Cruz, CA). The mouse monoclonal IgG1 antibody to CBP was raised against a peptide corresponding to amino acids 2422–2441 of CBP of human origin (Santa Cruz Biotechnology, Santa Cruz, CA).

### Reporter gene constructs and luciferase expression assays

Luciferase reporter plasmids were constructed to contain the U3 region of an FeLV LTR containing one, two or three copies of the 21-bp element. The U3 region of the FeLV-A/61E LTR, containing one copy of the 21-bp element, was cloned into the firefly luciferase reporter plasmid pGL2-Basic (Promega Corp., Madison, WI). The LTR was then substituted between *Pst*I and *Hinc*II restriction sites with homologous sequences from a naturally occurring LTR containing two 21-bp elements [Chandhasin *et al*., manuscript submitted] or from FeLV-945, which contains three 21-bp elements. Luciferase reporter plasmids were also constructed in which point mutations were introduced into either the first or second c-Myb binding site in the 21-bp triplication of the FeLV-945 LTR. Binding site mutants were constructed by designing synthetic oligonucleotides **-/+ **(5'-GCTGAAACAGCAGAAGTTTCAAGGCCACTGCCAGCAG**A**TTCAAGGCCACTGCCAGCAGTTTCAAGGCCACTGCCAGCAGTCTCCAGGCTCCCCAGTTGAC-3') and **+/- **(5'-GCTGAAACAGCAGAAGTTTCAAGGCCACTGCCAGCAGTTTCAAGGCCACTGCCAGCAG**A**TTCAAGGCCACTGCCAGCAGTCTCCAGGCTCCCCAGTTGAC-3') that contained a point mutation in the first or second binding site, respectively (mutated base indicated by boldface and underline). The indicated point mutation had previously been shown to ablate c-Myb binding [16]. A double-stranded form of each sequence was generated using the filling primer (5'-GAACTCTGGTCAACTGGGGAGCCTGGAGACTGCTG-3') and the Klenow fragment of DNA polymerase. The resulting double stranded oligonucleotides were digested with *AluI/HincII *and substituted into the LTR of FeLV-A/61E. The *KpnI/PstI *fragment of the resulting recombinant LTR was then excised and cloned into the pGL2-Basic luciferase reporter plasmid. Finally, a luciferase reporter plasmid was developed that contained the isolated 21-bp triplication cloned upstream of the SV40 promoter in pGL2-Promoter (Promega Corp., Madison, WI). A double-stranded DNA fragment containing the 21-bp triplication was generated by PCR amplification using the oligonucleotide GS945 (described above) as template and primers fw945-kpn1 (5'- GCTCGGTACCAGCTGAAACAGCAGAAGTTTC) and rv945-sac1 (5'- ATGCTGAGCTCAACTGGGGAGCCTGGAGACT). The resulting amplification product was digested with *KpnI/SstI *and inserted into the multiple cloning site upstream of the SV40 promoter in the reporter plasmid.

For reporter gene assays, 2 × 10^5 ^cells were seeded in triplicate into 6-well tissue culture plates. The next day, reporter plasmids (500 ng) were introduced into cultured cells by lipid-mediated transfection (Lipofectamine Plus; Invitrogen, Carlsbad, CA) in the presence of pRL-TK (5 ng) in a 100:1 ratio. pRL-TK encodes *Renilla *luciferase and was used as an internal control for transfection efficiency. Firefly and Renilla luciferase activities were quantified 24 hours later using the Dual-Luciferase Reporter Assay System (Promega Corp., Madison, WI). Data from triplicate wells were analyzed statistically using one-way ANOVA and Bonferroni post test. Statistical significance was considered as p < 0.05. In some assays, the c-Myb expression vector FL-Myb (described above) was added to the transfection in increasing amounts (50 ng – 500 ng). The 5XMRE plasmid was used in reporter gene assays as a positive control. This plasmid contains five tandem c-Myb binding sites cloned upstream of a luciferase gene (a gift of Dr. Linda Wolff, National Cancer Institute).

### Virus Replication Assay

5 × 10^5 ^K-562 cells, uninfected or chronically infected with recombinant FeLVs GA-945L and GA-61EL (described above) were seeded in triplicate into 24-well tissue culture plates. A full-length CBP expression vector (4 μg; a gift from Dr. Matthew Burow, Tulane University Medical School) was introduced into the cells by lipid mediated transfection using Lipofectamine 2000 reagent (Invitrogen, Carlsbad, CA). Culture supernatants were collected three days later and reverse transcriptase activity was quantified as previously described [8]. Data from triplicate wells were analyzed statistically using one-way ANOVA and Bonferroni post test. Statistical significance was considered as p < 0.05.

## Competing interests

None declared.

## Authors' contributions

SLF developed reporter gene constructs and performed binding assays, gene expression and virus replication assays. SP identified and initially demonstrated c-Myb binding sites. KRR developed the reporter gene assays. LSL directed the experimental design, implementation and interpretation of data. All authors read and approved the final manuscript.
